# Skin Anti-Aging Potential through Whitening and Wrinkle Improvement Using Fermented Oil Derived from *Hermetia illucens* Larvae

**DOI:** 10.3390/ijms25052736

**Published:** 2024-02-27

**Authors:** Dooseon Hwang, Tae-Won Goo, Seung Hun Lee, Eun-Young Yun

**Affiliations:** 1 Department of Integrative Biological Sciences and Industry, Sejong University, Seoul 05006, Republic of Korea; 2 Department of Biochemistry, School of Medicine, Dongguk University, Gyeongju 38766, Republic of Korea

**Keywords:** anti-aging, fermentation, *Hermetia illucens* larvae oil, whitening, wrinkle improvement

## Abstract

As the aging population increases, so has interest among emerging seniors in anti-aging ingredients that enhance functionality by incorporating fermentation with natural materials. In this study, fermentation conditions for enhancing the functionality of *Hermetia illucens* larvae oil (HIO) were established, and its anti-aging potential was evaluated. First, the lipase activity and amount of lipid degradation products of the fermentation strains were measured in order to select *Lactobacillus gasseri* and *Lactiplantibacillus plantarum* as the strains with high fermentation ability. A fermentation period of 28 d and a fermentation method that uses only the strain culture medium were established by evaluating the fermentation degree after fermenting HIO with the selected strains. The whitening functionality test results of fermented HIO (FHIO) showed an increase of approximately 20% in extracellular tyrosinase inhibition activity compared with HIO. Additionally, within melanocytes, there was a 12% increase in tyrosinase inhibition activity and a 26% enhancement in melanin production inhibition ability. For wrinkle-improving functionality, it was observed that, for fibroblasts, there was a 10% increase in collagen production, a 9% increase in collagenase inhibition ability, and an 8% increase in elastase inhibition ability. Therefore, FHIO was confirmed to be an effective cosmetic raw material, with high functionality for anti-aging within the senior generation. This is achieved through increased whitening and wrinkle-improving functionality.

## 1. Introduction

The cosmetics industry, which includes anti-aging cosmetics, is becoming an increasingly elderly-friendly industry [1]. Cosmetics are now used not only for simple functions, such as skincare and cleanliness, but also as tools for enhancing individuals’ psychological stability and self-esteem [2,3,4,5,6]. Therefore, there is a demand for the development of cosmetics that can maintain beauty even as people age, fueled by the increasing average lifespan and the economic power of the baby boomer generation [7]. However, there are issues with existing synthetic ingredients, such as allergic contact dermatitis, skin irritation, phototoxicity, and photoallergic reactions [8,9]. Additionally, concerns persist regarding the adverse effects of the synthetic ingredients used in anti-aging cosmetics. An example of these is hydroquinone, which is a key ingredient in whitening cosmetics and has been classified as a carcinogen [10]. As a result, there is growing interest in the research and development of highly functional natural cosmetic ingredients that are both effective and safe, particularly those related to the prevention of skin aging, such as through whitening and wrinkle improvement [11]. Natural cosmetic ingredients are reported to have fewer side effects, lower irritation, faster skin absorption, and higher biodegradability compared with synthetic ingredients [1,11,12,13]. Consequently, research efforts have been ongoing to discover anti-aging cosmetic ingredients from various natural sources [12,13]. However, most efforts to find natural resources focus on plants, and research generally involves the production of extracts to find functional ingredients. While using natural ingredients is highly desirable, there are drawbacks, including the sustainability of plant resources [14]. Furthermore, due to the Nagoya Protocol, difficulties, such as securing price competitiveness and supply delays, arise when sourcing resources from foreign organisms [15].

Animal-derived ingredients have been reported to effectively improve various skin issues in terms of hydration, elasticity, and wrinkles, as they possess similar structures to humans and so facilitate the rapid and safe absorption of active substances [16,17,18,19]. Mucus from the *Huso huso*, a sturgeon, has been reported to promote cell proliferation and wound healing, effectively regenerating and moisturizing damaged skin [16]. Byproducts such as oil from sheep, horses, and donkeys have been found to prevent skin moisture loss, providing moisturizing and protective effects [17,18,19]. Additionally, they contain selenium, conferring antioxidant properties, promoting cell proliferation, and exhibiting antimicrobial effects [19]. Animal-derived ingredients offer the advantage of sustainability due to their utilization of byproducts [20]. However, they have the disadvantage of being less popular among consumers due to ethical issues arising from the exploitation of animals.

*Hermetia illucens* (black soldier fly) larvae, a representative feed insect, feed on organic waste. This is not only very economical but, as the animal is an invertebrate, also incurs relatively few ethical issues. In addition, in the process of manufacturing *Hermetia illucens* larvae as feed ingredients, they undergo a defatting process, producing oil as a byproduct [20,21,22]. *Hermetia illucens* larvae oil (HIO) is known to be rich in lauric acid, oleic acid, and linoleic acid, which play significant roles in the barrier function of human skin (Table 1) [20,23,24,25].

Meanwhile, fermenting cosmetic raw materials has the advantage of reducing particle size, increasing skin absorption, and reducing toxicity through the adsorption of harmful substances by microorganisms. In addition, it has the advantage of improving functionality by increasing the content of active substances such as whitening, anti-inflammatory, and anti-aging through secondary metabolites produced by microorganisms [26,27].

Therefore, in this study, we attempted to confirm the anti-aging functionality of HIO, which had not previously been researched, through skin whitening and wrinkle improvement functions, and whether the functionality of fermented HIO (FHIO) is increased to confirm its potential as a material for senior-friendly cosmetics.

## 2. Results

### 2.1. Evaluation of the Lipid Fermentation Abilities in 10 Selected Strains

Ten strains were reported to have high activity for lipase, a lipid-degrading enzyme, and they were ranked in the following order: *Lactobacillus gasseri*, *Lactiplantibacillus pentosus*, *Lactiplantibacillus plantarum*, *Lactobacillus curvatus*, *Pediococcus pentosaceus*, *Staphylococcus warneri*, *Pseudomonas pseudoalkaligensis*, *Pediococcus acidiloctici*, and *Saccaromyces cerevisiae* (Table 2). Among these, *L. gasseri* exhibited the significantly highest lipase activity. The measurement of a lipid-degradation product, free glycerol, showed that the levels were in the following order: *L. gasseri*, *L. plantarum*, *L. pentosus*, and *L. curvatus*, with the significantly highest free glycerol production in *L. gasseri* and *L. plantarum*. Another degradation product, free fatty acid, was higher in *L. gasseri*, *P. pentosus*, *L. plantarum*, and *S. warneri*, with statistically significantly higher levels found in *L. gasseri* (Table 3). Therefore, based on the high activities of the degradation enzyme and the high production of degradation products, *L. gasseri* and *L. plantarum* were selected as the strains for HIO fermentation.

### 2.2. Evaluation of the Fermentation Conditions of Hermetia illucens Oil Fermented with Selected Strains

After culturing the two selected strains, the fermentation process for HIO was evaluated over a period of 0 to 35 d using two methods: one utilizing only the culture medium without the strains after incubation, and the other using the strains with the culture medium. As the fermentation time progressed for both methods, the quantity of fermentation products increased until day 28, after which no further increase was observed (Table 4). After 28 d of fermentation, the free glycerol content and free fatty acid in the HIO were measured, and a significantly higher amount was determined when using only the culture medium than when fermenting with the culture medium and the strains (Table 4). Therefore, a method was established in which fermentation was conducted for 28 d using only the medium in which the strain was cultured.

### 2.3. Evaluation of Cytotoxicity for the Determination of Solvent Concentration and Hermetia illucens Oil Treatment Concentration in Melanoma Cells and Fibroblasts

**Figure 1:**
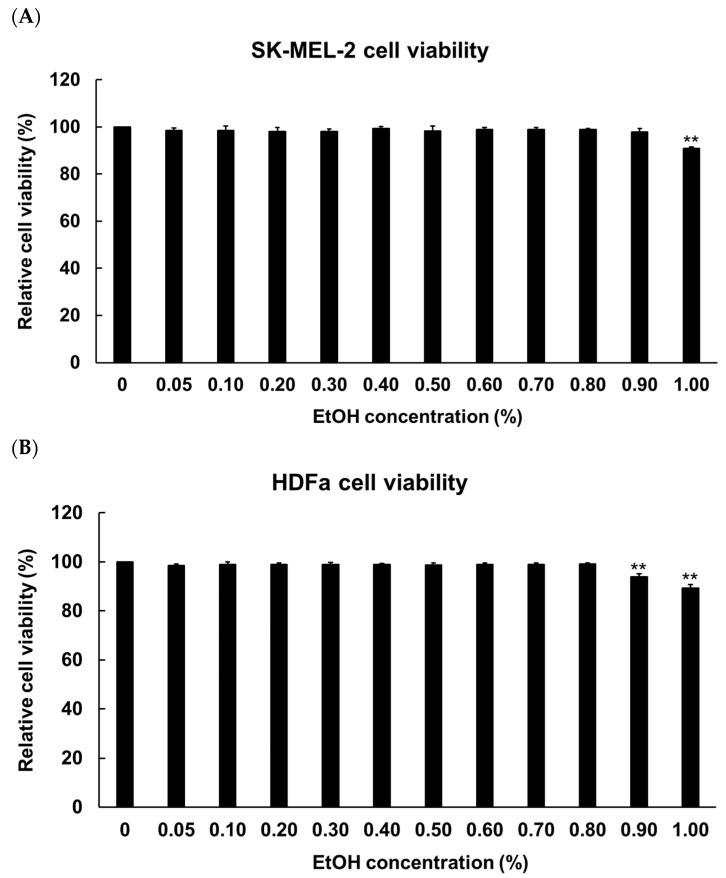
SK-MEL-2 (**A**) and HDFa (**B**) cell viability according to ethanol concentration. Comparisons between 0 and each EtOH concentration were performed using Student’s *t*-test. ** *p* < 0.01 indicates a significant difference between the control and each EtOH concentration. SK-MEL-2, human melanoma cell line; HDFa, human dermal fibroblast.

**Figure 2:**
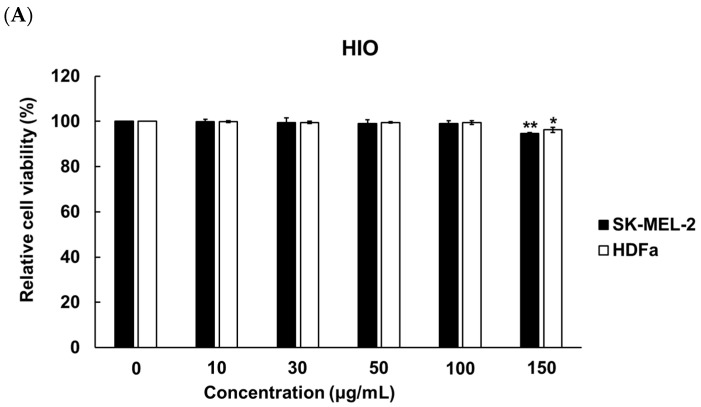

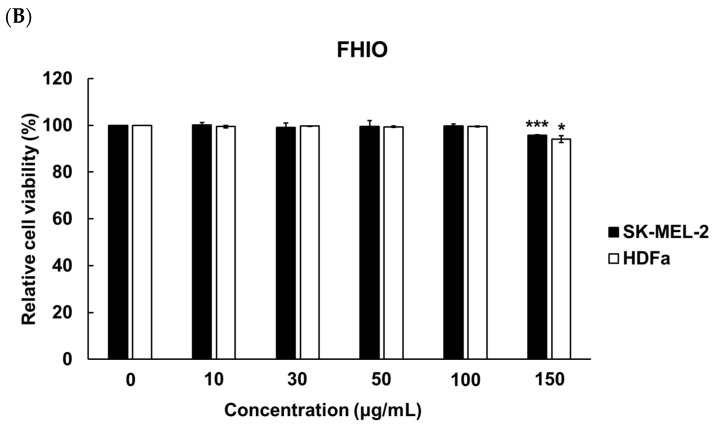
Cell viabilities according to the use of HIO (**A**) and fermented HIO (FHIO) (**B**). The values represent the mean ± standard deviation of triplicate experiments. Comparisons between 0 and each concentration were performed using Student’s *t*-test. * *p* < 0.05, ** *p* < 0.01 and *** *p* < 0.001 indicate a significant difference between the control and each oil concentration. HIO, *Hermetia illucens* larvae oil; FHIO, fermented *Hermetia illucens* larvae oil; SK-MEL-2, human melanoma cell line; HDFa, human dermal fibroblast.

To determine the concentration of ethanol for solubilizing HIO, fermented HIO (FHIO), and fatty acids, melanocyte (SK-MEL-2 cells) and fibroblast (human dermal fibroblast, HDFa) cells were treated with 0 to 1.00% concentrations of ethanol (EtOH). The results showed a significant decrease in cell viability at EtOH concentrations of 0.1% for SK-MEL-2 cells and 0.09% for HDFa cells (Figure 1). To establish the maximum non-toxic concentration for cell treatment with HIO and FHIO, the SK-MEL-2 cells and HDFa cells were treated with concentrations ranging from 0 to 150 μg/mL for 48 h. The findings revealed a significant reduction in cell viability when the concentration exceeded 100 μg/mL for both types of oil (Figure 2). Therefore, HIO and FHIO were dissolved in 0.8% EtOH at a concentration of 100 μg/mL for use in this study.

### 2.4. Evaluation of the Skin-Whitening Potential of Hermetia illucens Oil and Fermented Hermetia illucens Oil in Melanoma Cells

**Figure 3:**
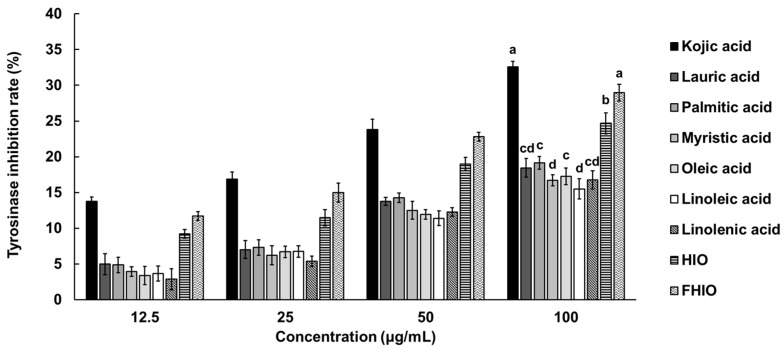
In vitro tyrosinase inhibition rates of single fatty acids, HIO, and FHIO. The value of the negative control group was 0.014 ± 0.005%. The values represent the mean ± standard deviation of triplicate experiments. Different superscripts above the bars indicate significant differences as determined by Duncan’s multiple range test. HIO, *Hermetia illucens* larvae oil; FHIO, fermented *Hermetia illucens* larvae oil.

To confirm the degree of inhibition of tyrosinase activity involved in melanin synthesis, the in vitro tyrosinase inhibition rate of HIO, FHIO, and single fatty acids were compared with that of kojic acid. As a result, concentration-dependent tyrosinase inhibition rates were confirmed in all treatment groups, with HIO and FHIO exhibiting higher inhibition rates than those of individual fatty acids. FHIO demonstrated a significantly higher inhibition rate of 30.78% compared to HIO’s 26.73%, suggesting that fermentation increases the tyrosinase inhibition activity of HIO (Figure 3).

**Figure 4:**
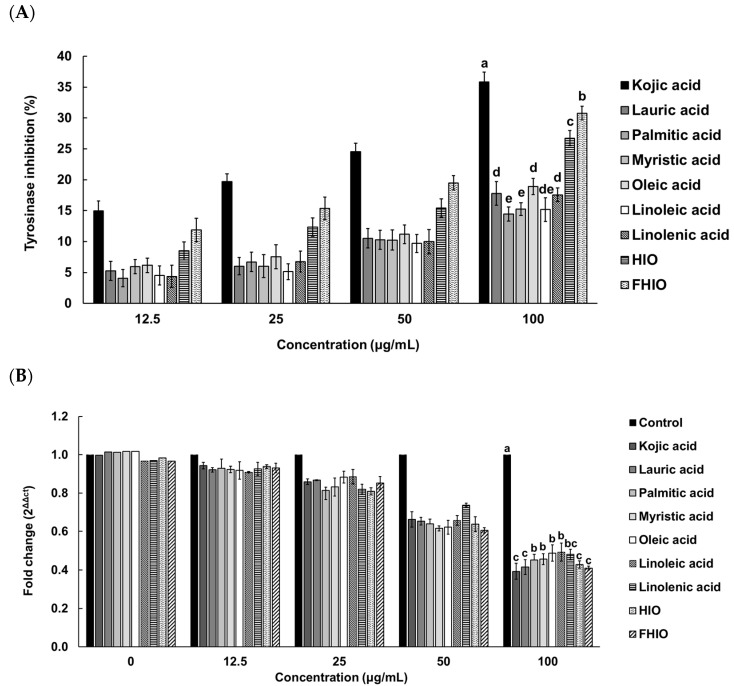
Inhibition rates of single fatty acids, HIO, and FHIO for the tyrosinase activity (**A**) and tyrosinase mRNA level (**B**) in SK-MEL-2 cells. The value of the negative control group in (**A**) was 0.057 ± 0.032%. The values are reported as the mean ± standard deviation of triplicate experiments. Different superscripts above the bars indicate significant differences as determined by Duncan’s multiple range test. HIO, *Hermetia illucens* larvae oil; FHIO, fermented *Hermetia illucens* larvae oil; SK-MEL-2, human melanoma cell line.

**Figure 5:**
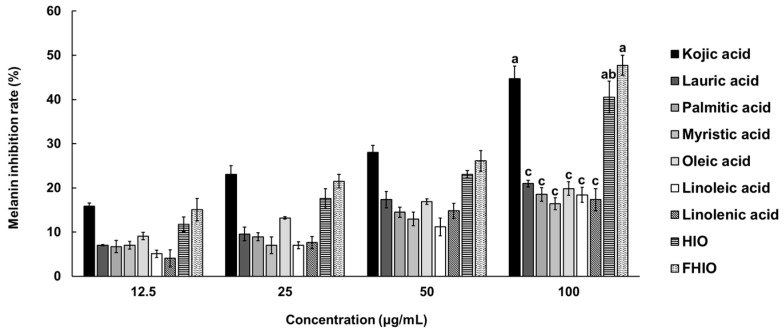
Inhibition rates of single fatty acids, HIO, and FHIO for melanin production in SK-MEL-2 cells. The value of the negative control group was 0.007 ± 0.002%. The values are reported as the mean ± standard deviation of triplicate experiments. Different superscripts above the bars indicate significant differences as determined by Duncan’s multiple range test. HIO, *Hermetia illucens* larvae oil; FHIO, fermented *Hermetia illucens* larvae oil; SK-MEL-2, human melanoma cell line.

To further evaluate the extent of the tyrosinase inhibition potential of HIO and FHIO, SK-MEL-2 cells were treated with HIO, FHIO, and single fatty acids for 48 h. As a result, the two types of insect oil showed higher tyrosinase inhibition rates than that of single fatty acids. FHIO (28.69%) showed a significantly higher inhibition rate than that of HIO (24.68%), which confirmed that fermentation increases the tyrosinase inhibition rate, and the rate was significantly similar to that of kojic acid (32.58%) (Figure 4A). In addition, HIO and FHIO were confirmed to reduce the mRNA expression level of tyrosinase by 0.428 and 0.410 times, respectively, and the level of FHIO was significantly similar to that of kojic acid (0.393-fold) (Figure 4B). Since tyrosine is oxidized by tyrosinase to synthesize melanin, the skin-whitening potential of HIO and FHIO was also confirmed by the rate of inhibition of melanin production. The results showed that the melanin inhibition rates of HIO, FHIO, and single fatty acids are concentration-dependent. In particular, FHIO exhibited a significantly increased rate of melanin inhibition at 47.75% compared to HIO (40.53%), and this rate was notably similar to that of kojic acid (44.73%) (Figure 5). Therefore, it was confirmed that HIO had greater whitening potential after fermentation; in SK-MEL-2 cells treated with FHIO, its ability to inhibit tyrosinase, reduce the mRNA expression of tyrosinase, and inhibit melanin production were confirmed to be significantly similar to those of kojic acid.

### 2.5. Evaluation of the Skin Wrinkle Improvement Potential of Hermetia illucens Oil and Fermented Hermetia illucens Oil in Fibroblasts

**Figure 6:**
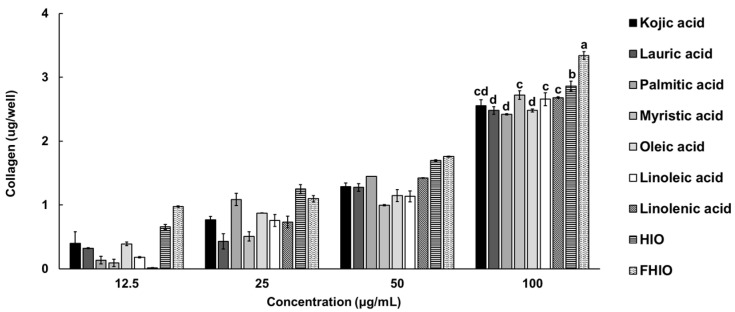
Collagen production ability in HDFa cells for single fatty acids, HIO, and FHIO. The value of the negative control group was 1.105 ± 0.095 μg/well. The values are reported as the mean ± standard deviation of triplicate experiments. Different superscripts above the bars indicate significant differences as determined by Duncan’s multiple range test. HDFa, human dermal fibroblast; HIO, *Hermetia illucens* larvae oil; FHIO, fermented *Hermetia illucens* larvae oil.

**Figure 7:**
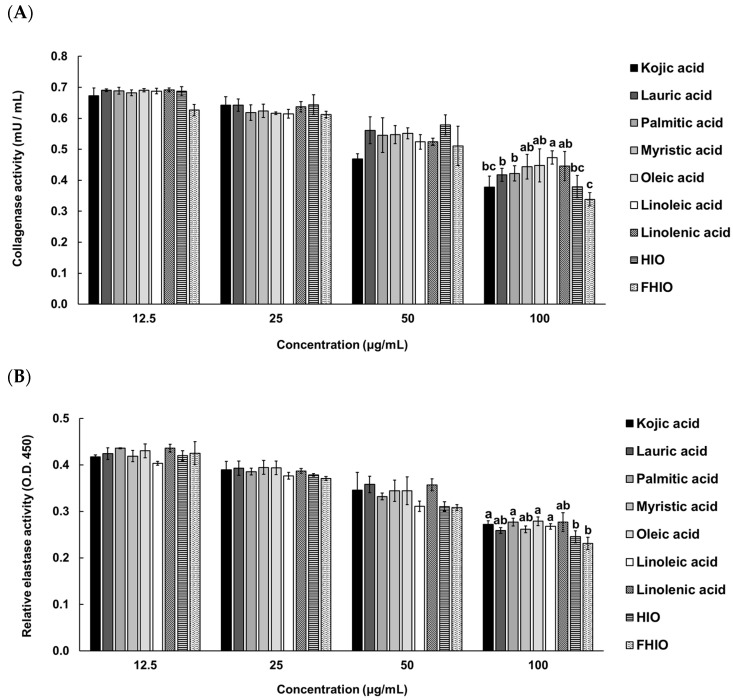
Collagenase (**A**) and elastase (**B**) activities in HDFa cells of single fatty acids, HIO, and FHIO. The value of the negative control group in (**A**) was 0.721 ± 0.065 mU/mL. The values are reported as the mean ± standard deviation of triplicate experiments. Different superscripts above the bars indicate significant differences as determined by Duncan’s multiple range test. HDFa, human dermal fibroblast; HIO, *Hermetia illucens* larvae oil; FHIO, fermented *Hermetia illucens* larvae oil.

To confirm whether HIO and FHIO promote collagen production in the skin, the amount of collagen was measured after treating fibroblasts. As a result, it was confirmed that the amount of collagen in the fibroblasts increased in a concentration-dependent manner in the treatment groups, except for the control. Additionally, the treatments with HIO and FHIO resulted in significantly greater amounts of collagen than that in the treatment with single fatty acids. In particular, in the case of FHIO (3.342 μg/well), the amount of collagen significantly increased in comparison with that in the HIO treatment (2.862 μg/well), showing that fermentation increases the efficacy of HIO in promoting collagen production (Figure 6). To further confirm the wrinkle improvement effect, the activity of collagenase, a collagen hydrolyzing enzyme, was measured. The collagenase activity of the fibroblasts treated with HIO and FHIO was 0.379 mU/mL and 0.338 mU/mL, respectively, which was lower than that of single fatty acids (Figure 7A). The collagenase inhibition activity of FHIO was confirmed to be significantly superior to that of HIO. Lastly, the amount of elastase, an enzyme that decomposes elastin, which is a protein that maintains skin elasticity, was compared according to OD 450 values. As a result, HIO and FHIO were found to have significantly lower elastase activity than that of single fatty acids, and FHIO (0.231) had a lower OD 450 value than that of HIO (0.245) (Figure 7B). Considering the above results, the fermentation of HIO increases collagen production and decreases collagenase and elastase activity in fibroblasts, thus confirming its superior wrinkle improvement efficacy.

## 3. Discussion

As the elderly population continues to grow, the “silver industry” is also experiencing trends of growth [1,2,3,4,5,6,7]. While it varies from country to country, the baby boomer generation, which was characterized by a rapid increase in birth rates, has undergone similar experiences of rapid industrialization, economic growth, and the development of mass consumer culture worldwide, leading to an aging population [1,7]. In aging countries, the baby boomer generation is transitioning into an elderly consumer group, driving the development and activation of products and services related to the “silver industry” [2,5]. Recently, fermentation-utilizing microorganisms and enzymes have been reported to enhance both the safety and activity of cosmetic ingredients [26]. This is because fermentation breaks down substances into smaller sizes to increase their absorption by the skin and removes harmful substances through microbial degradation or adsorption [27]. In this study, we aimed to develop cosmetic materials for the elderly population by exploring the anti-aging functionality of HIO, which is rich in lauric acid, oleic acid, and linoleic acid, and fermenting HIO to enhance its functionality.

In the evaluation of the fermentation abilities of various strains, *L. gasseri* and *L. plantarum* were found to have excellent fermentation abilities. Therefore, although conditions for fermentation with these two strains were established in this study, additional research is needed to determine why these two strains have excellent fermentation abilities. The fermentation products of HIO fermented with the two strains did not increase further after 28 d. This indicated that the amount of fermentation enzyme did not continuously increase when a certain amount of medium containing microorganisms was added. This is thought to be because the number of bacteria no longer increased but decreased after the exponential phase. When only the culture medium was used, it was confirmed that the amount of decomposition products no longer increased because the amount of enzymes decreased or became inactive over time. In addition, microorganisms secrete lipase for metabolism; in the media containing microorganisms, the decomposition products of HIO were used for the microorganisms’ metabolism, so the fermentation efficiency was thought to be lower than when only the medium was used.

The inhibition of tyrosinase, a major oxidative enzyme in melanin synthesis, was examined in FHIO [28,29,30,31,32,33]. FHIO inhibited tyrosinase activity more effectively in both test tubes and within cells compared to HIO and individual fatty acids. Therefore, FHIO was shown to inhibit melanin synthesis by suppressing the activity of tyrosinase, the key enzyme in melanin synthesis. This is believed to be due to fatty acids such as lauric acid, oleic acid, and linoleic acid being isolated during the fermentation process, transforming them into a more absorbable form and exhibiting a synergistic effect, thus resulting in improved whitening functionality.

Collagen is the main component of connective tissue, and it accounts for about 90% of the dermis layer of the skin [7]. Hyaluronic acid, which binds with moisture, is associated with collagen, so increasing the collagen content of the skin leads to improved elasticity and the prevention or reduction of wrinkles [7,11]. Here, it was found that the level of collagen synthesis increased more in cells treated with FHIO than in those treated with single fatty acids. Additionally, FHIO was shown to decrease the activity of collagenase, an enzyme that breaks down collagen. As skin aging progresses, the level of collagen synthesis decreases, and the expression of metallopeptidase inhibitor 1, which inhibits collagenase, also decreases, leading to an increase in the collagen degradation rate [7]. Therefore, FHIO is considered to delay skin aging by increasing collagen production and inhibiting collagenase activity. Elastin, another major component of connective tissue alongside collagen, plays a role in supporting collagen fibers firmly when they are combined [34,35]. It is a protein associated with skin elasticity, and an increase in the amount of elastase, which breaks down elastin, has been reported to loosen the skin barrier and accelerate skin aging. FHIO and HIO significantly reduced the amount of elastase in comparison to single fatty acids by increasing the durability of elastin and potentially preventing skin aging.

In conclusion, when fermented using only the culture medium of two types of microorganism, FHIO was found to decrease the mRNA expression level of tyrosinase, a key enzyme in the melanin synthesis pathway in melanocytes. This resulted in a reduction in tyrosinase activity, thus potentially inhibiting the synthesis of melanin that could have been deposited in the skin and thereby confirming its skin-whitening efficacy. Additionally, FHIO was found to increase the production of collagen involved in fibroblast binding and to decrease the amounts of collagenase and elastase, thus indicating its efficacy in improving skin wrinkles. Therefore, FHIO was confirmed as an effective and sustainable animal-derived cosmetic ingredient, capable of improving skin aging in the elderly population through its skin whitening and wrinkle improvement effects.

## 4. Materials and Methods

### 4.1. Hermetia illucens Oil, Microbial Strains, Culture Conditions, and Fermentation Efficacy Assays of Different Strains

HIO was provided by Greenteko (Hwaseong-si, Republic of Korea), and the extraction method was as follows: The larvae used for oil extraction were dried in an 18 kW conveyor microwave oven for 4 min. HIO was obtained by extracting 1 kg of larvae for 20 min at 120 °C using a continuous high-temperature expeller oil press (PJ-010, Poongjin, Pyeongtaek, Republic of Korea). The microorganisms that were evaluated for fermentation efficacy were the *L. gasseri* (KACC 12424), *L. pentosus* (KACC 12428), *L. plantarum* (KACC 12404), *L. curvatus* (KACC 12415), *B. subtilis* (KACC 19623), *P. pseudoalcaligenes* (KACC 10119), *P. acidilactici* (KACC 12307), *S. warneri* (KACC 10785), *P. pentosaceus* (KACC 12311), and *S. cerevisiae* (KACC 30008) strains, and they were obtained from the Korean Agricultural Collection (KACC, Wanju-gun, Republic of Korea). The fermentation capacity of each strain was measured with only the supernatant, which was acquired through centrifugation (4 °C, 8935× *g*, 10 min) after incubating the strains at appropriate temperatures and in media appropriate for each strain for 18 h. The activities of lipase, free fatty acids, and free glycerol for each strain were measured using the Lipase Activity Assay Kit (MAK046, Sigma-Aldrich, St. Louis, MO, USA), Free Glycerol Kit (ab65337, abcam, Cambridge, UK), and Free Fatty Acid Quantitation Kit (MAK044, Sigma-Aldrich, St. Louis, MO, USA), respectively, according to the manufacturers’ instructions.

### 4.2. Analysis of Fermentation Efficacy According to Fermentation Period and Fermentation Method for Hermetia illucens Oil

The bacterial culture media for the fermentation were prepared in two ways. The first was to culture *L. gasseri* and *L. plantarum* in MRS medium (69966, Sigma-Aldrich, St. Louis, MO, USA) for 48 h at 37 °C and then use both the bacteria and the culture medium (strains with culture medium). The second was to use only the medium from which the bacteria were removed through centrifugation (4 °C, 8935× *g*, 10 min) after the same 48 h of culturing (only culture medium). For fermentation, 2 g of the strains with the culture medium and only the culture medium were mixed with 20 g of HIO, and then fermented in a shaking incubator (37 °C to 200 rpm). Fermentation was carried out in a glass tube for 0–35 d, and after completion, the oil and layered medium were removed with a micropipette. The HIO after fermentation was evaluated for the content of free glycerol and free fatty acids, which are products of lipid decomposition. Measurements were performed according to the protocols enclosed in the Free Glycerol Kit (ab65337, abcam, Cambridge, UK) and Free Fatty Acid Quantitation Kit (MAK044, Sigma-Aldrich, St. Louis, MO, USA), respectively.

### 4.3. Cell Culture and Cell Viability Analysis for a Selection of HIO Solvents and HIO Treatment Concentrations

Melanocytes (SK-MEL-2) and human fibroblast cells (HDFa) were purchased from the American Type Culture Collection (ATCC, Manassas, VA, USA). The cells were incubated at 37 °C in a 5% carbon dioxide atmosphere with DMEM (LM001-11, WELGENE, Seoul, Republic of Korea) containing 1000 mg/L glucose, 10% FBS, and 1% antibiotic–antimycotic (AA) (Life Technology, Carlsbad, CA, USA). When cell saturation reached 80% confluence, the cells were suspended in trypsin, plated at 7 × 10^4^ cells per well of a 6-well plate, and incubated for 24 h. Afterward, the media were exchanged with 2 mL of DMEM containing 10% FBS, 1% AA, and ethanol, HIO, FHIO, or single fatty acids. Each well was covered with parafilm to prevent vaporization and incubated for 48 h. After incubation, the cells and media were divided through centrifugation (558× *g*, 5 min) and applied to all the experiments in this study. Cell viability was measured using a CellTiter 96^®^ AQueous One Solution Cell Proliferation Assay kit (G3582, Promega, Madison, WI, USA).

### 4.4. Analysis of the Skin-Whitening Effects of HIO and FHIO

#### 4.4.1. In Vitro Tyrosinase Inhibition Rate

The in vitro tyrosinase inhibition rate was determined using a Tyrosinase Activity Assay Kit (MAK395, Sigma-Aldrich, St. Louis, MO, USA) according to the manufacturer’s instructions.

#### 4.4.2. Tyrosinase Inhibition Rate and mRNA Expression Level in SK-MEL-2 Cells

The total RNA of the SK-MEL-2 cells was isolated using TRIzol^®^ Reagent (15596026, Invitrogen, Waltham, MA, USA), and cDNA was synthesized using a High Capacity cDNA Reverse Transcription Kit (4368814, Applied Biosystems, Foster City, CA, USA). The tyrosinase inhibition rate in the SK-MEL-2 cells was determined using a tyrosinase Activity Assay Kit (MAK395, Sigma-Aldrich, St. Louis, MO, USA) according to the manufacturer’s manual. The tyrosinase mRNA level was measured using BrightGreen 2× qPCR MasterMix-No Dye (Applied Biological Materials, Richmond, BC, Canada), according to the supplier’s manual. Glyceraldehyde 3-phosphate dehydrogenase (GAPDH) was used as an endogenous control, and the relative gene expression levels were analyzed using the 2^−ΔΔCt^ method. Primers for GAPDH (forward 5′-GAGTCAACGGATTTGGTCGT-3′, reverse 5′-GATCTCGCTCCTGGAAGATG-3′) and TYRP1 (forward 5′- ATGGCAGAGATGATCGGGAG-3′, reverse 5′-GAGCTTCAACTCCAACCCTT-3′) were used for cDNA amplification [36]. The thermocycling conditions were: 95 °C for 3 min followed by 40 cycles of 95 °C for 15 s, 60 °C for 30 s, and 72 °C for 30 s.

#### 4.4.3. Melanin Inhibition Rate in SK-MEL-2 Cells

Lysis of the SK-MEL-2 cells was performed in radio immunoprecipitation assay (RIPA) buffer (R2002, Biosesang, Yongin-si, Republic of Korea). After cell lysis, melanin was analyzed using Human Melatonin ELISA Kit (CSB-E14051h, Cusabio, Houston, TX, USA) according to the manufacturer’s instructions.

### 4.5. Analysis of Skin Wrinkle Improvement Effect of HIO and FHIO

Lysis of the HDFa cells was performed in RIPA buffer (R2002, Biosesang, Yongin-si, Republic of Korea). After cell lysis, the activities of collagen, collagenase, and elastase were measured using the Total Collagen Assay Kit (NBP2-59748, Novus Biological, Centennial, CO, USA), Collagenase Activity Assay Kit (ab196999, abcam, Cambridge, UK), and Human Elastase ELISA Kit (NBP2-82489, Novus Biological, Centennial, CO, USA), respectively, according to the manufacturers’ instructions.

### 4.6. Statistical Analysis

All experiments were performed in triplicate, and the results are presented as the mean ± standard deviation. A test of the normality of the data was performed using the Kolmogorov–Smirnov test. Comparisons between two groups were performed using Student’s *t*-test, and comparisons of three or more groups were performed using Duncan’s multiple range test. SPSS ver. 18 (SPSS Inc., Chicago, IL, USA) was used for the analysis.

## 5. Conclusions

In this study, the anti-aging potential of HIO and FHIO was evaluated to examine their use as raw materials for cosmetics. HIO and FHIO were confirmed to have anti-aging potential through their skin-whitening and wrinkle improvement effects. Furthermore, it was confirmed that FHIO had an enhanced anti-aging effect in comparison to that of HIO due to its fermentation. Therefore, FHIO has high economic efficiency because it uses byproducts of insect processing, does not cause ethical problems because its source is an invertebrate, and has more value than HIO as an elderly-friendly cosmetic material with improved functionality.

## Figures and Tables

**Table 1:** Fatty acid profile of the *Hermetia illucens* larvae used in this study.

	Content (%)
Capric acid (C10:0)	0.7
Lauric acid (C12:0)	23.6
Myristic acid (C14:0)	6
Myristoleic acid (C14:1)	0.1
Palmitic acid (C16:0)	20.3
Palmitoleic acid (C16:1)	2.7
Margaroleic acid (C17:0)	0.5
Stearic acid (C18:0)	4.6
Oleic acid (C18:1)	23.2
Linoleic acid (C18:2)	15.9
Linolenic acid (C18:3)	2.2
Arachidic acid (C20:0)	0.2
Saturated fatty acids	55.9
Monounsaturated fatty acid	26.0
Polyunsaturated fatty acid	18.1

**Table 2:** Lipid-degrading enzyme activity of 10 microorganism strains.

Microorganisms	Lipase (mU/mL)
*L. gasseri*	3.879 ± 0.397 ^a^
*L. pentosus*	3.557 ± 0.031 ^b^
*L. plantarum*	3.240 ± 0.329 ^c^
*L. curvatus*	3.135 ± 0.003 ^c^
*B. subtillis*	2.910 ± 0.048 ^e^
*P. pseudoalcaligenes*	3.065 ± 0.310 ^cd^
*P. acidiloctici*	3.037 ± 0.062 ^cd^
*S. warneri*	3.077 ± 0.021 ^d^
*P. pentosaceus*	3.122 ± 0.061 ^c^
*S. cerevisiae*	3.036 ± 0.046 ^d^

The values represent the mean ± standard deviation of triplicate experiments. Different superscripts within a column indicate significant differences as determined by Duncan’s multiple range test.

**Table 3:** Free glycerol and free fatty acid product levels of the 10 microorganism strains.

	Free Glycerol (μmol/mL)	Free Fatty Acid (μmol/mL)
*L. gasseri*	1.330 ± 0.075 ^a^	2.915 ± 0.175 ^a^
*L. pentosus*	1.094 ± 0.054 ^b^	2.396 ± 0.136 ^cd^
*L. plantarum*	1.297 ± 0.069 ^a^	2.508 ± 0.145 ^b^
*L. curvatus*	1.083 ± 0.062 ^b^	2.378 ± 0.027 ^c^
*B. subtillis*	1.014 ± 0.009 ^c^	2.403 ± 0.009 ^c^
*P. pseudoalcaligenes*	1.063 ± 0.022 ^b^	2.297 ± 0.021 ^d^
*P. acidiloctici*	1.047 ± 0.010 ^c^	2.377 ± 0.021 ^c^
*S. warneri*	1.077 ± 0.001 ^b^	2.440 ± 0.001 ^b^
*P. pentosaceus*	1.019 ± 0.009 ^d^	2.710 ± 0.013 ^ab^
*S. cerevisiae*	0.951 ± 0.011 ^e^	2.365 ± 0.053 ^c^

The values represent the mean ± standard deviation of triplicate experiments. Different superscripts within a column indicate significant differences as determined by Duncan’s multiple range test.

**Table 4:** Content of free glycerol and free fatty acid products in *Hermetia illucens* oil (HIO) according to fermentation method and fermentation period.

		Free Glycerol (μmol/mL)		Free Fatty Acid (μmol/mL)	
Fermentation Method		Strains with Culture Medium	Culture Medium Only	Strains with Culture Medium	Culture Medium Only
Fermentation period (days)	0	2.678 ± 0.030 ^d^	2.780 ± 0.032 ^d^	3.138 ± 0.069 ^d^	3.122 ± 0.071 ^d^
	0.5	2.705 ± 0.143 ^d^	2.807 ± 0.040 ^cd^	3.148 ± 0.287 ^d^	3.133 ± 0.040 ^d^
	1	2.860 ± 0.023 ^d^	2.864 ± 0.019 ^cd^	3.170 ± 0.052 ^d^	3.255 ± 0.019 ^cd^
	2	2.795 ± 0.051 ^cd^	2.889 ± 0.041 ^cd^	3.211 ± 0.048 ^d^	3.295 ± 0.041 ^cd^
	3	2.863 ± 0.053 ^cd^	2.967 ± 0.091 ^cd^	3.307 ± 0.057 ^cd^	3.292 ± 0.091 ^cd^
	4	3.041 ± 0.169 ^cd^	3.145 ± 0.075 ^c^	3.429 ± 0.039 ^c^	3.314 ± 0.074 ^cd^
	5	3.387 ± 0.280 ^cd^	3.294 ± 0.159 ^c^	3.441 ± 0.048 ^c^	3.526 ± 0.159 ^c^
	7	3.685 ± 0.114 ^c^	3.990 ± 0.124 ^b^	3.632 ± 0.027 ^b^	3.917 ± 0.124 ^b^
	14	3.668 ± 0.280 ^b^	4.071 ± 0.480 ^ab^	4.215 ± 0.065 ^a^	4.799 ± 0.480 ^a^
	21	3.745 ± 0.114 ^a^	4.091 ± 0.231 ^a^	4.240 ± 0.048 ^a^	4.824 ± 0.231 ^a^
	28	3.816 ± 0.098 ^a^	4.098 ± 0.058 ^a^	4.251 ± 0.027 ^a^	4.855 ± 0.058 ^a^
	35	3.728 ± 0.120 ^a^	4.031 ± 0.069 ^a^	4.214 ± 0.065 ^a^	4.798 ± 0.069 ^a^

The values represent the mean ± standard deviation of triplicate experiments. Different superscripts within a column indicate significant differences as determined by Duncan’s multiple range test.

## Data Availability

Data is contained within the article.
